# The worldwide holoparasitic Apodanthaceae confidently placed in the Cucurbitales by nuclear and mitochondrial gene trees

**DOI:** 10.1186/1471-2148-10-219

**Published:** 2010-07-21

**Authors:** Natalia Filipowicz, Susanne S Renner

**Affiliations:** 1 Systematic Botany and Mycology, University of Munich (LMU), Menzinger Strasse 67, Munich, Germany

## Abstract

**Background:**

Of the c. 450 families of flowering plants, only two are left "unplaced" in the most recent APG classification of angiosperms. One of these is the Apodanthaceae, a clade of c. 19 holoparasitic species in two or three genera occurring in North and South America, Africa, the Near East, and Australia. Because of lateral gene transfer between Apodanthaceae and their hosts it has been difficult to infer the family's true closest relatives.

**Results:**

Here we report a phylogenetic analysis of 16 accessions representing six species of Apodanthaceae from the United States, Chile, Iran, and Australia, using the mitochondrial *matR *gene and the nuclear 18S gene. Data matrices include 190 *matR *sequences from up to 95 families in 39 orders of flowering plants and 197 18S sequences from 101 families representing the 16 orders of rosids. Analyses were performed at the nucleotide and at the amino acid level. Both gene trees agree with angiosperm phylogenies found in other studies using more genes. Apodanthaceae and the seven families of the order Cucurbitales form a clade with 100% bootstrap support from *matR *and 56% from 18 S. In addition, the Apodanthaceae and Cucurbitales *matR *gene sequences uniquely share two non-synonymous codon changes and one synonymous change, as well as a codon insertion, already found by Barkman et al. (2007).

**Conclusions:**

Apodanthaceae belong in the Cucurbitales with which they share inferior ovaries, parietal placentation and a dioecious mating system, traits that are ancestral in Cucurbitales and which can now be interpreted as possible synapomorphies of an enlarged order Cucurbitales. The occurrence of Apodanthaceae in the Americas, Africa, the Near East, and Australia, and their adaptation to distantly related host species in the Fabaceae and Salicaceae suggest a long evolutionary history.

## Background

Among the c. 450 families of angiosperms [1] are about 11 parasitic lineages [2] of which some have completely lost the ability to photosynthesize. Such non-photosynthetic parasites grow embedded within the host tissues (as endoparasites) and emerge only during sexual reproduction [3]. Molecular-phylogenetic studies in recent years have revealed the sister groups of most parasitic plants [e. g. [2,4-10]. Only the Apodanthaceae and the likewise holoparasitic Cynomoriaceae have not yet been placed with confidence, and the Apodanthaceae are also the only unplaced family-level clade in the latest classification of flowering plants [1,11]. Before the advent of large-scale molecular sequencing and until about 2004, Apodanthaceae were considered to belong in the order Rafflesiales, together with Rafflesiaceae, Cytinaceae and Mitrastemonaceae [12,13], mainly based on few or solitary, rather large to very large flowers with a single set of tepals that are commonly united into a conspicuous, corolloid calyx.

The aerial portions of Apodanthaceae consist of very short flowering shoots, each with a single flower that bursts out of the host's cortex during development [13]. Flowers are less than a centimeter across. The family contains three genera: the neotropical *Apodanthes *with about five species from Panama throughout South America to Uruguay and Argentina; the disjunctly distributed *Pilostyles *with c. nine species in the southern United States, Mexico, Panama, Venezuela, and Brazil, one species in Iran and Syria, and three in southwestern Australia [14]; and the African *Berlinianche *(a genus name that has never been validly published) with one or two species in Angola, southern Congo, Zambia, and Zimbabwe, Malawi, and Tanzania. Both species of "*Berlinianche*" were originally described in *Pilostyles*, and seven of the species of *Pilostyles *and *Apodanthes *have been moved between these genera, suggesting that the generic distinctions are not clear-cut. *Apodanthes *is supposed to have deciduous perianth segments, while *Pilostyles *has persistent perianth segments. The three genera also overlap in their host preferences, with *Apodanthes *parasitizing the Fabaceae *Adesmia *and the Salicaceae *Casearia *and *Xylosma*, and *Pilostyles *and *Berlinianche *parasitizing a range of other Fabaceae [13,15].

Two molecular-systematic studies have included Apodanthaceae together with representative samples of other flowering plants [2,16]. Both also included the parasites' host species to check for possible horizontal gene transfer between parasite and host. Nickrent et al. [16] sequenced the mitochondrial (mt) gene *atp1 *for *Apodanthes caseariae*, *Berlinianche aethiopica*, and *Pilostyles thurberi*, mt *matR *for *A. caseariae *and *P. thurberi*, and nuclear ribosomal 18S for *P. thurberi*. The *atp1 *gene placed the three genera together, but showed *Pisum *(Fabaceae) and *Polemonium *(Ericales) embedded among them, albeit without statistical support. The *matR *gene placed *Apodanthes *and *Pilostyles *together (posterior probability 100) and both as the sister clade to *Begonia *and *Cucurbita *(Cucurbitales) with a posterior probability of 98. The nuclear 18S region, finally, placed *P. thurberi *as sister to *Gossypium *and *Pavonia *(Malvales), albeit again without statistical support.

Barkman et al. [2] included only *P. thurberi*, but sequenced three mt genes, *atp1*, *matR*, and *cox1*, for a data set that covered at least one family from 44 of the 45 orders of angiosperm. A maximum likelihood tree from the combined data grouped *P. thurberi *as sister to *Begonia *and *Echinocystis *(the two representatives of the order Cucurbitales), although with low bootstrap support. The relationship received support from a seemingly uniquely shared 3 bp (one codon) insertion in the *matR *genes of *P. thurberi, Begonia *and *Echinocystis*.

Both studies [2,16] explained the contradictory placements of Apodanthaceae in the *atp1*, *matR*, and *18S *gene trees as resulting from horizontal gene transfer (HGT). This is a plausible explanation because plant mitochondrial genes are sometimes exchanged between species [17-20], and most known cases of HGT come from parasitic plants, which have permanent tissue contact with their hosts [8,16,21-24]. Even nuclear DNA can be transferred between parasites and their hosts [25]. Specifically, Nickrent et al. [16] regarded the *matR *and *atp1 *tree topologies as resulting from HGT. The only placement accepted as trustworthy was that of *P. thurberi *near Malvales in the 18S tree. By contrast, Barkman et al. [2] suggested that *matR *was not affected by HGT, but that *atp1 *had recently been transferred from a Fabaceae host species to *P. thurberi*.

Barkman et al.'s [2] evidence for this interpretation was twofold. First, analyses of *atp1 *suggested that four endoparasite lineages, *Cytinus, Mitrastema, Pilostyles*, and *Rafflesia*, all were closely related to their respective hosts, which appeared extremely unlikely and suggested that *atp1 *might be prone to HGT. Second, when comparing the *atp1 *sequences of two accessions of *P. thurberi*, one from Arizona (AZ), one from Texas (TX), Barkman et al. noted that the sequences differed at eight sites in the gene's central region. In contrast, comparisons of *atp1 *from *Pilostyles-*AZ to *atp1 *from its host revealed identical sequences for 804 bp in the central region, but 15 differences at the 5' and 3' ends. Based on these and other comparisons, Barkman et al. concluded that *Pilostyles-*AZ and *Pilostyles-*TX *atp1 *represent chimeric xenologs that result from multiple horizontal gene transfers. Chimeric *atp1 *genes in Apodanthaceae were also inferred from the transfer of a 79-nt track of chloroplast (cp) DNA to the mitochondria in *Apodanthes caseariae*, but not *Pilostyles thurberi *[26]. The only functional cp sequences so far recovered from any Apodanthaceae come from the small subunit of the plastid ribosomal RNA 16S gene [14,27]. These results on chimeric sequences indicate that *atp1 *is unsuited for investigating the phylogenetic relationships of Apodanthaceae.

Other mitochondrial genes that might be explored for the purpose of phylogenetically placing the Apodanthaceae are the mt *cox1 *gene [2] and the *nad1 *B/C exons, e.g. [20]. The *cox1 *gene, however, has an extremely low substitution rate, limiting its usefulness for large-scale phylogenetics [28], and the *nad1 *B/C region is prone to lateral gene transfer [8,20]. Nevertheless, the present study also explored these genes for Apodanthaceae phylogenetics.

The most promising genetic markers, however, clearly are the nuclear 18S region and the mt *matR *gene. The highly conserved 18S gene codes for RNA, a major subunit of the ribosome, and large 18S alignments have been found to contain sufficient signal to recover many known angiosperm family relationships [29-31]. Also, Apodanthaceae 18S trees are required to test the surprising Apodanthaceae/Malvales clade discovered by Nickrent et al. [16]. The maturase-related (*matR*) gene is retained regardless of photosynthetic ability, making it useful for parasite phylogenetics [2,6,9,16]. Different from the *cox1 *intron [28], no case of *matR *loss has come to light [32]. *MatR *sequences are now available for 700+ flowering plants, and the gene's phylogenetic utility in rosids has been shown in a critical study [33].

We therefore generated 18S and *matR *sequences from multiple accessions and species of Apodanthaceae from the United States, Chile, Iran, and Australia. The sequences were aligned with representatives of all seven families of Cucurbitales, an order comprising 111 genera with about 2450 species [34]: Anisophylleaceae (29-40 species in 4 gen.), Begoniaceae (1400 spp. in 2 gen.), Coriariaceae (15 spp. in 1 genus), Corynocarpaceae (6 spp. in 1 genus), Cucurbitaceae (950 spp. in c. 100 gen.), Datiscaceae (2 spp. in 1 genus), and Tetramelaceae (2 spp. in 2 gen.). Following Zhu et al. [33], we performed amino acid-level analyses and constructed the DNA alignment according to the amino acid alignment. Such codon-based alignments have been shown to result in better statistical support by reducing misalignment of non-homologous regions [33,35,36]. As explained above, we also explored the utility of *cox1 *and *nad1 *B/C as potential markers for placing Apodanthaceae within angiosperms.

## Results

### Phylogenetics

**Figure 1:**
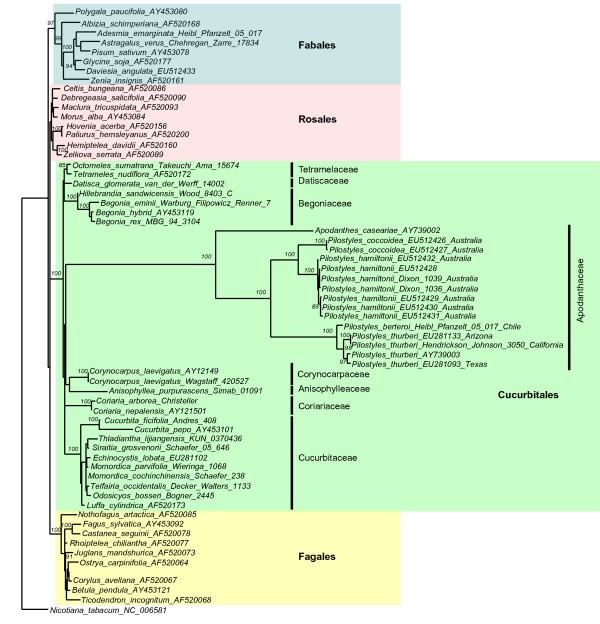
**Maximum likelihood phylogeny obtained from the matR codon (nucleotide) alignment for 64 Cucurbitales, Rosales, Fagales, and Fabales**. Numbers above branches indicate ML bootstrap support >75%.

Figure 1 shows a maximum likelihood (ML) tree from the codon alignment of 64 of the 190 *matR *sequences analyzed in this study (see Additional File 1 for the full 190-taxon tree and Additional File 2 for a tree from the amino acid alignment). The included sequences represent 95 families from 39 of the c. 60 orders of flowering plants, plus 16 accessions from six species of Apodanthaceae from the United States, Chile, and Australia. The rosid orders Fagales, Rosales, and Cucurbitales are each recovered as monophyletic (the Fagales and Cucurbitales with 100% bootstrap support, the Rosales with 80% for codon alignment). The inclusion of Apodanthaceae in Cucurbitales has 100% bootstrap support at the nucleotide level (Figure 1) and 95% on amino acid level (Additional File 2). Family relationships remain unresolved. *Apodanthes *is sister to *Pilostyles*, and accessions of the West Australian species *P. coccoidea *and *P. hamiltonii *form the sister clade to the North American *P. thurberi *(represented by four accessions) and the Chilean *P. berteroi*. We were unable to amplify the *matR *gene from several samples of the Iranian *P. haussknechtii*, even with four sets of universal primers.

**Figure 2:**
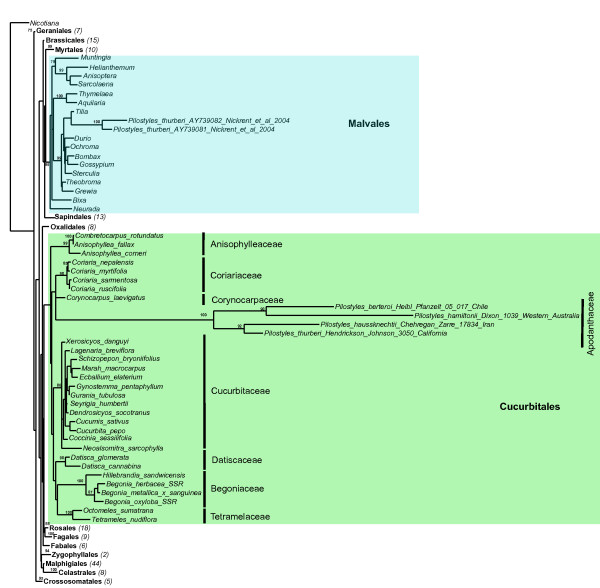
**Maximum likelihood phylogeny obtained from the 18S nucleotide matrix for 197 rosid taxa**. Numbers above branches indicate ML bootstrap support >75%. Numbers in parentheses following the orders' names indicate numbers of genera included in the analysis.

Figure 2 shows an ML tree from the 18S matrix of 197 angiosperm species from 101 families in 16 orders of rosids, including representatives of all families of Cucurbitales and four species of Apodanthaceae from the United States, Iran, Chile, and Australia. Within Cucurbitales, the Apodanthaceae form the sister clade to Coriariaceae and Corynocarpaceae, albeit with low bootstrap support. Within the family, Iranian *P. haussknechtii *is sister to North American *P. thurberi*, while South American *P. berteroi *is sister to the Australian *P. hamiltonii*. The two 18S sequences of *P. thurberi *from GenBank [16] fall within Malvales as a sister clade to *Tilia*, as found in the original study.

An alignment of 143 *cox1 *exon sequences (from Cusimano et al. [28]) that included "*Berlinianche*" *aethiopica *and *Pilostyles thurberi *sequences from GenBank [2] placed the latter on an extremely long branch near *Viola, Linum*, and *Malpighia*, albeit without statistical support (data not shown). A single Apodanthaceae *nad1 *B/C sequence could not be successfully aligned with the 70-taxon *nad1 *B/C data set of Won and Renner [20] nor with newly generated Cucurbitales *nad1 *B/C sequences.

### Codon substitutions

All Apodanthaceae and Cucurbitales share three unique codon changes: at positions 256, a C changed to a T, and at positions 505 and 700, a G to an A (coordinates from *Nicotiana tabacum *NC006581). Two of these changes are non-synonymous, and the change at position 700 also results in changing the polarity of the encoded amino acid (alanine to threonine). In addition, there is synonymous and variable substitution at position 532; *Apodanthes caseariae *and the Australian Apodanthaceae (*P. hamiltonii *and *P. coccoidea*) here have a codon CUG, while the remaining Cucurbitales have CUC. Both codons encode leucine. American *Pilostyles *species at this position have AUG, encoding methionine, while Fagales, Fabales and Rosales have AUC, coding for isoleucine. We also found the codon insertion uniquely shared by Cucurbitales and Apodanthaceae (at position 288, coordinates from *Nicotiana tabacum *NC006581), previously reported for *P. thurberi*, *Begonia*, and *Echinocystis *(at position 341, coordinates from *Arabidopsis thaliana *NC001284; [2]). Australian Apodanthaceae and *A. caseariae *moreover share a 12-nt insertion (corresponding to 4 amino acids) at position 1531.

## Discussion

### Phylogenetic placement of the Apodanthaceae in the Cucurbitales

The mitochondrial and nuclear data analyzed here show that Apodanthaceae belong in the Cucurbitales, an order here represented by 22 or 29 species in 16 or 22 genera from its seven families (Figures 1 and 2). In both trees, all sequences of Apodanthaceae form a clade, and all families of Cucurbitales are recovered as monophyletic. The 18S tree moreover recovers the core cucurbit clade of Datiscaceae, Begoniaceae, Tetramelaceae, and Cucurbitaceae, the Corynocarpaceae and Coriariaceae clade, and the placement of Anisophylleaceae also found with 12,000 nucleotides of nuclear, plastid and mitochondrial gene sequences [34]. While not resolving within-Cucurbitales family relationships, *matR *is sufficiently variable to recover angiosperm ordinal relationships that agree with those found with other kinds of data [1,37-39]. An example is that Cucurbitales are sister to Fagales as found by Zhang et al. [34] and Moore et al. [39].

Nickrent et al. [16] were the first to find a relationship between *Pilostyles *and Cucurbitales using *matR*, but distrusted this grouping (as well as their *atp1 *tree) because of possible HGT. Our data reject this explanation because Apodanthaceae accessions from the different hosts and continents group together, which is implausible under the assumption of HGT. Another argument against HGT affecting our data sets is that the nuclear and mitochondrial trees agree in their placement of Apodanthaceae. It was only when Barkman et al. [2] found a codon insertion in the *matR *gene that was uniquely shared by *Pilostyles, Echinocystis *and *Begonia *that a possible phylogenetic relationship among these taxa became a strong hypothesis. The wider taxon sampling used here confirms the presence of a synapomorphic codon insertion in all Cucurbitales (including Apodanthaceae) at position 288 in *Nicotiana tabacum *NC006581. Additionally, we found three other codon changes shared by Apodanthaceae and Cucurbitales, two of them non-synonymous. This fits with Zhu et al.'s [33] finding that the *matR *non-synonymous substitution rate is relatively high and close to the gene's synonymous substitution rate, indicating relaxed selection.

### Morphological traits supporting the Cucurbitales/Apodanthaceae clade and possible relationships with Coriariaceae and Corynocarpaceae

The Cucurbitales are a large order (7 families, 111 genera, 2450 species), characterized by ancestrally inferior ovaries and parietal placentation [34,40], traits than can now be interpreted as a morphological synapomorphy with Apodanthaceae, especially since parietal placentation is a rare feature. Other traits characterizing most Cucurbitales are unisexual flowers, and a dioecious mating system [34].

Apodanthaceae also have strictly unisexual flowers, with dioecy very common [14,41]. However, unisexual flowers and dioecy are strongly overrepresented among heterotrophic angiosperms [42], cautioning against overconfidence in this trait as evolutionarily conservative.

The 18S tree (Figure 2) shows the Apodanthaceae in a polytomy with Coriariaceae (a family of one genus and 15 species) and Corynocarpaceae (one genus with 6 species), albeit without statistical support. These families are sister clades [34] and differ from other Cucurbitales in having superior ovaries with apical placentation and mostly bisexual flowers ([34] for a tabulation of Cucurbitales morphological traits). Within Cucurbitales, they are therefore morphologically distant from Apodanthaceae.

## Conclusions

The fossil record of Cucurbitales goes back to the Uppermost Paleocene and Lower Eocene London Clay [34], indicating the deep evolutionary history of the order. So far, no Cucurbitales are known to be hosts of Apodanthaceae, and irrespective of the latter's precise placement in Cucurbitales, it is clear that Apodanthaceae have been on their own evolutionary trajectory for a very long time. The family's presence in both, South America and Africa, might point to a history going back to a time when some of the fragments of the super-continent Gondwana were still close to each other. However, analysis of the biogeography of Apodanthaceae will require inclusion of the two African species, one only known from the type collection destroyed during World War II. The genetically and morphologically well-supported inclusion of Apodanthaceae in the Cucurbitales solves one of the last family-level "problems" in flowering plant phylogenetics [1] and finally allows assessing which genetic material these holoparasites may have acquired from host species.

## Methods

### Taxon sampling

The *matR *matrix included 190 sequences, representing 95 families from 39 orders of angiosperms; 22 of the sequences newly generated for this study. The 18S matrix included 197 sequences representing 101 families from 16 orders of rosids; four new Apodanthaceae sequences were generated. For detailed information on voucher material, host species and GenBank accession numbers see additional file 3.

### Molecular methods

Molecular methods, including DNA extraction, PCR amplification, and DNA sequencing were performed as described in Zhang et al. [34]. *MatR *primers used where those of Barkman et al. [9], and 18S primers were those of Nickrent and Starr [43].

### Alignments and phylogenetic analyses

We downloaded the *matR *alignment of Zhu et al. [33] (TreeBase accession number M3533), extracted 30 core rosid sequences from it and added 34 sequences relevant to this study, namely all available and new sequenced representatives of Apodanthaceae, the seven families of Cucurbitales, five of the eight families of Fagales, five families of the nine families of Rosales, and two of the four families of Fabales, including Apodanthaceae host species. DNA sequences were then translated using the ExPASy Proteomics Server [44] and aligned at the amino acid level using MAFFT [45,46]. The corresponding DNA codon alignment (reverse-translated) was then produced using the PAL2NAL program [47]. The 64-taxon-matrix created generated by PAL2NAL did not include ambiguously aligned sections.

We also produced a 190-taxon *matR *matrix, which included additional eudicots, using MAFFT and followed by minor manual adjustments. Regions of uncertain alignment in the 190-taxon-matrix were excluded prior to analysis (161 nt from the beginning,76 nt from the end and 7 nt fragment at position 814 of original alignment).

The 567-taxon three-gene alignment of Soltis et al. [30,31] was received from P. Soltis, the matrix then reduced to the 18S gene (~1800 bp) of the rosids to which were added the 18S sequences of Cucurbitales from Zhang et al. [34] and the newly generated Apodanthaceae sequences. This resulted in 197-taxon 18S matrix.

Phylogenetic analyses for the *matR *gene were performed at the nucleotide (codon) and the amino acid level, and for the 18S gene at the nucleotide level, using ML and full ML bootstrapping (100 replicates), with the GTR + GAMMA substitution model for the nucleotide alignment and the JTT model for the AA alignment, as implemented in RAxML [48]. The *matR *64-taxon tree and the 18S 197-taxon tree were rooted on *Nicotiana tabacum*, and the *matR *190-taxon tree on Austrobaileyales.

## Authors' contributions

NF generated sequences and alignments, performed data analyses, and worked on the manuscript. SSR conceived the study, obtained material, and drafted the manuscript.  Both authors read and approved the final manuscript.  

## Authors' information

NF is currently a postdoc in SSR's group and has a permanent position in the Department of Biology and Pharmaceutical Botany of the Medical University of Gdansk (Poland), from where she was granted a leave-of-absence to pursue this study.

## Acknowledgements

The authors thank H. Schaefer for several Cucurbitales matR sequences, S. Zarre, C.  Heibl, and K. Dixon for material from Iran, Chile, and Australia, A. Fleischmann and  M. Gottschling for helpful discussions, T. Barkman, C. Davis, and D. Nickrent for  their constructive reviews, and the associate editor, S. Mathews, for cross-checking  information.

## Supplementary Material

Additional file 1 **Maximum likelihood phylogeny obtained from *matR *sequences representing 190 species from 95 families of flowering plants**. Numbers above branches indicate ML bootstrap support >75%. Click here for file

Additional file 2 **Maximum likelihood phylogeny obtained from the *matR *amino acid matrix for 64 Cucurbitales, Rosales, Fagales, and Fabales**. Click here for file

Additional file 3 **Sources of plant material and GenBank accession numbers used in this study**. Click here for file
