# Supplementary figures and images for: The worldwide holoparasitic Apodanthaceae confidently placed in the Cucurbitales by nuclear and mitochondrial gene trees
Source: BMC Evol Biol. 2010 Jul 21;10:219. doi: 10.1186/1471-2148-10-219 (PMC3055242; doi:10.1186/1471-2148-10-219)

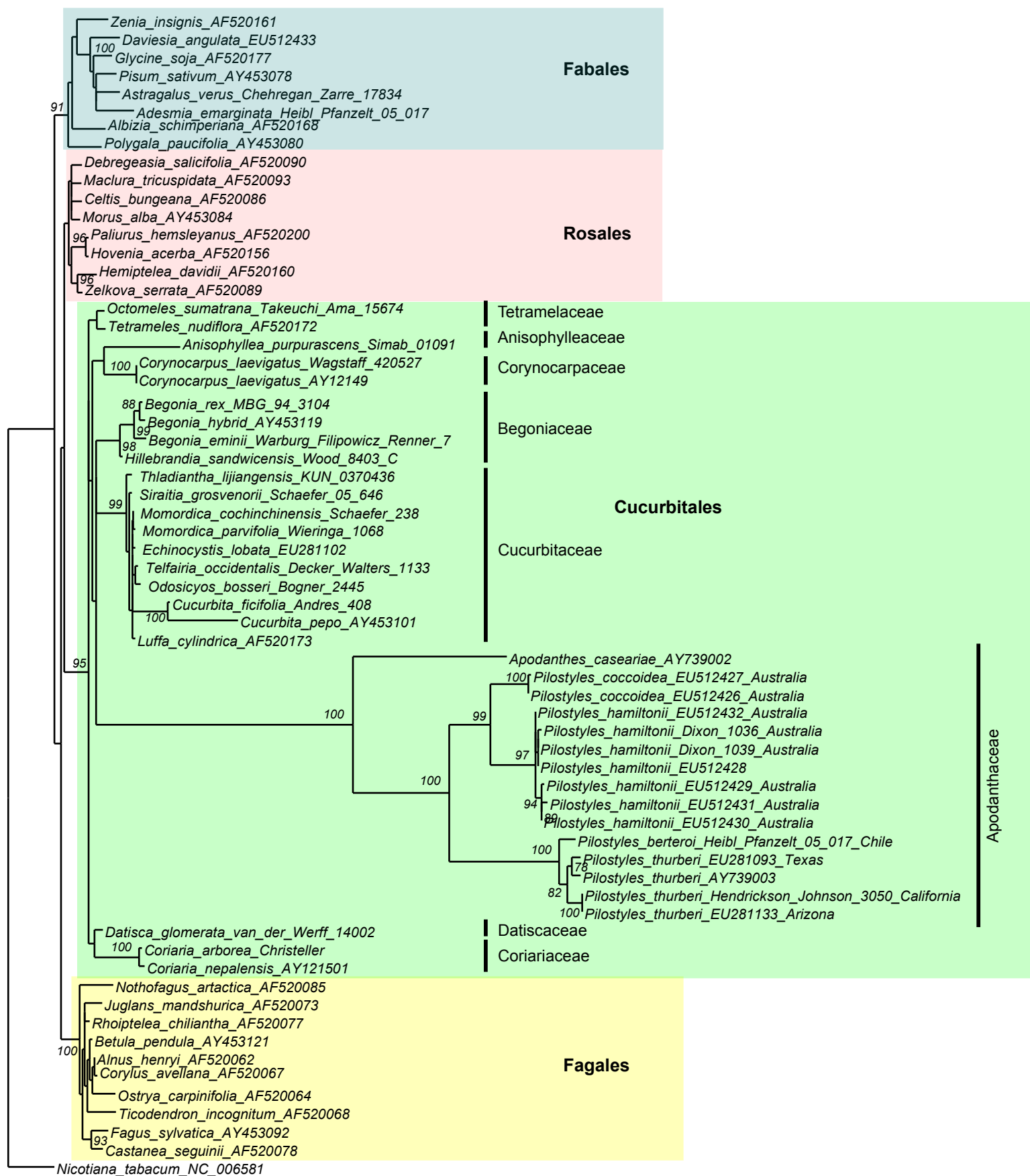

Supplement: Additional file 2 — Maximum likelihood phylogeny obtained from the matR amino acid matrix for 64 Cucurbitales, Rosales, Fagales, and Fabales. [file 1471-2148-10-219-S2.PDF]
