# Supplementary material for: The worldwide holoparasitic Apodanthaceae confidently placed in the Cucurbitales by nuclear and mitochondrial gene trees
Source: BMC Evol Biol. 2010 Jul 21;10:219. doi: 10.1186/1471-2148-10-219 (PMC3055242; doi:10.1186/1471-2148-10-219)
Supplement: Additional file 3 — Sources of plant material and GenBank accession numbers used in this study. [file 1471-2148-10-219-S3.PDF]

Species Name	Voucher/Origin	matR	18S
**Apodanthaceae**			
Apodanthes caseariae Poit.	Costa Rica: D. Nickrent 3007 (SIU), parasitic on parasitic on *Casearia* sp.	AY739002 [1]	–
Pilostyles berteroi Guill. = *Apodanthes berteroi* (Guill.) Gardn.	Chile: C. Heibl & S. Pfanzelt 05-017 (M), parasitic on *Adesmia emarginata*	HM600776 (this study)	HM592263 (this study)
Pilostyles coccoidea K.R. Thiele	Australia: Badgingara, 3-1 (PERTH), parasitic on *Jacksonia floribunda*	EU512426 [2]	–
Pilostyles coccoidea	Australia: Badgingara, 2-1 (PERTH), parasitic on *Jacksonia floribunda*	EU512427 [2]	–
Pilostyles hamiltonii C.A. Gardn.	N/A	EU512429 [2]	–
Pilostyles hamiltonii	Australia: Badgingara, 212 (PERTH), parasitic on *Daviesia angulata*	EU512430 [2]	–
Pilostyles hamiltonii	Australia: Cataby, 305 (PERTH), parasitic on *Daviesia angulata*	EU512431 [2]	–
Pilostyles hamiltonii	Australia: Kalamunda, DS28 (PERTH), parasitic on *Daviesia preissii*	EU512432 [2]	–
Pilostyles hamiltonii	Australia: near West Perth, K. Dixon 1039 (PERTH), parasitic on *Daviesia angulata*	HM600777 (this study)	HM592264 (this study)
Pilostyles hamiltonii	Australia: near West Perth, K. Dixon 1036 (PERTH), parasitic on *Daviesia angulata*	HM600778 (this study)	–
Pilostyles haussknechtii Boiss.	Iran: A. Chehregan & S. Zarre 17834 (M), parasitic on *Astragalus verus*	–	HM592265 (this study)
Pilostyles thurberi A. Gray	USA: California, L. Hendrickson & P. Johnson 3050 (MO), parasitic on *Psorothamnus* (*Dalea*) *emoryi*	HM600779 (this study)	HM592266 (this study)
Pilostyles thurberi	USA: Texas, D. Nickrent 4342 (SIU), parasitic on *Psorothamnus emoryi*	AY739003 [1]	AY739082 [1]
Pilostyles thurberi	USA: Texas, B. L. Turner 99-270 (TEX), parasitic on *Psorothamnus emoryi*	EU281093 [3]	–
Pilostyles thurberi	USA: California, Nickrent 2766 (SIU), parasitic on *Psorothamnus emoryi*	–	AY739082 [1]

Pilostyles thurberi	USA: Arizona, C. W. DePamphilis 2004.0A (PAC), parasitic on *Psorothamnus emoryi*	EU281133 [3]	–
**Anisophylleaceae**			
Anisophyllea corneri Ding Hou	Malaysia: S. FRI 40360 (KEP)	–	AY968390 [4]
Anisophyllea fallax S. Elliot	Madagascar: G. Schatz et al. 3808 (MO)	–	AY929365 [4]
Anisophyllea purpurascens Hutch. & Dalziel	Gabon: Simab 010918 (MO)	HM600767 (this study)	–
Combretocarpus rotundatus Danser	Indonesia: E. Mirmanto s.n. (BO)	–	AY968405 [4]
**Begoniaceae**			
Begonia eminii Warburg	Cameroon: Cult. Munich Botanical Garden, N. Filipowicz & S. S. Renner 7 (M)	HM600769 (this study)	–
Begonia hybrid	Cult., T. J. Barkman 364 (PAC)	AY453119 [1] and AY453119 [8]	–
Begonia herbacea Vell.	America: L. Forrest 163 (E)	–	AY968391 [4]
Begonia oxyloba Welw. ex Hook. f.	Africa: Hughes s.n. (L. Forrest 279) (E)	–	AY968392 [4]
Begonia rex Putz	Cult. Munich Bot. Gard. 94/3104	HM600770 (this study)	–
Hillebrandia sandwicensis Oliv.	USA: Hawaii, K. R. Wood 8403-C (PTBG)	HM600774 (this study)	AY968398 [4]
**Coriariaceae**			
Coriaria arborea Linds.	New Zealand: Palmerston North, J. Christeller s.n. (M)	HM600771 (this study)	–
Coriaria myrtifolia L.	Cult. UNC Chapel Hill, M. W. Chase 245 (NCU)	–	AF206891 [5]
Coriaria nepalensis Wall.	China: D.F. Chen 1 (no voucher)	AY121501 (unpublished)	AY968394 [4]
Coriaria ruscifolia L.	Mexico: M. Olson 836 (MEXU)	–	AY968395 [4]
Coriaria sarmentosa Forst. f.	New Zealand: Allan Herbarium, CHR 512491	–	AY968409 [4]
**Corynocarpaceae**			
Corynocarpus laevigatus J.R.Forst.&G.Forst.	New Zealand: Totara, near Thames, leg. S. Wagstaff (CHR acc. 420527)	HM600772 (this study)	–
Corynocarpus laevigatus	Cult. RBG Sydney: M.W. Chase 236 (NCU)	AY121499 (unpublished)	AF206892 [5]
**Cucurbitaceae**			

Coccinia sessilifolia (Sond.) Cogn.	Africa (cult. Mainz Bot Gard), S. S. Renner et al. 2763 (M)	–	AY973011 [4]
Cucurbita ficifolia Bouché	Ecuador: T. Andres 408 (NY)	HM008590 (this study)	
Cucurbita pepo L.	Cult., T. J. Barkman 371 (PAC)	AY453101 [1]	–
Cucurbita pepo L.	Cult., Soltis s.n. (WS)	–	AF206895 [5]
Cucumis sativus L.	Cult., Soltis s.n. (WS)	–	AF206894 [5]
Dendrosicyos socotranus Balf. f.	Mexico: M. Olson s.n. (MO)	–	AY968397 [4]
Ecballium elaterium (L.) A. Rich. ssp. *elaterium*	Mediterranean (cult. Mainz Bot Gard): S. S. Renner et al. 2768 (M)	–	AY973012 [4]
Echinocystis lobata (Michx.) Torr. & A. Gray	USA: Eastern US, J. R. McNeal 98.0805 (PAC)	EU281102 [3]	–
Gurania tubulosa Cogn. (syn.: *G. megistantha* J. D. Sm.)	South America (cult. Missouri Bot Gard): acc.1993-1657-4;	–	AY973013 [4]
Gynostemma pentaphyllum Makino	Japan: H. Takahashi 20712 (GIFU)	–	AY973014 [4]
Lagenaria breviflora (Benth.) Roberty	Ghana: M. Merello et al. 1331 (MO)	–	AY929371 [4]
Marah macrocarpus Greene	USA: Sonoran Desert, M. Olson s.n., 26 Nov, 2001 (MO)	–	AY973015 [4]
Momordica cochinchinensis Spreng.	China: H. Schaefer 238 (M)	HM008591 (this study)	–
Momordica parvifolia Cogn.	Gabon: J. J. Wieringa 1068 (WAG)	HM008592 (this study)	–
Neoalsomitra sarcophylla (Wall.) Hutchinson	Thailand (cult. Mainz Bot Gard): S. S. Renner et al. 2778 (M)	–	AY968399 [4]
Odosicyos bosseri Keraudren-Aymonin	Madagascar: J. Bogner 2445 (M)	HM008593 (this study)	–
Schizopepon bryoniifolius Maxim.	Japan: T. Fukuhara leg. seeds, cult. in St. Louis by S. Renner	–	AY968400 [4]
Seyrigia humbertii Keraudr.	Madagascar (cult. Missouri Bot Gard): acc. 1996-3485	–	AY973016 [4]
Siraitia grosvenorii (Swingle) C. Jeffrey ex Lu & Z. Y. Zhang	China: H. Schaefer 05/646 (M)	HM008594 (this study)	–
Telfairia occidentalis Hook.f.	Cameroon: D. Decker-Walters 1133 (FTG)	HM008595 (this study)	–
Thladiantha lijiangensis A. M. Lu & Z. Y. Zhang.	China: Chinese collector, KUN 0370436 (KUN)	HM008596 (this study)	–
Xerosicyos danguyi	Madagascar (cult. Mainz Bot	–	AY973017 [4]

Humbert	Gard): S. S. Renner *et al.* 2807 (M)		
**Datiscaceae**			
Datisca cannabina L.	Cult.: M. W. Chase 2745 (K)	–	AF008952 [5]
Datisca glomerata (Presl) Baill	USA: California, H. van der Werff 14002 (MO)	HM600773 (this study)	–
Datisca glomerata (Presl) Baill	USA: California, San Diego County, A. Liston 767 (RSA)	–	U42426 [6]
**Fabaceae (host species)**			
Adesmia emarginata Clos	Chile: C. Heibl & S. Pfanzelt 05-017 (M)	HM600766 (this study)	–
Astragalus verus Oliv.	Iran: A. Chehregan & S. Zarre 17834 (M)	HM600768 (this study)	–
**Tetramelaceae**			
Octomeles sumatrana Miq.	Papua New Guinea: W. Takeuchi & D. Ama 15674 (LAE)	HM600775 (this study)	–
Octomeles sumatrana	Sarawak: Lundu District, Gunung Gading, C. T. Philbrick & Ismai 2273 (RSA)	–	AF008953 [7]
Tetrameles nudiflora R. Br.	China: Xishuangbanna Botanical Garden, Q.J. Li 1302 (XSBN)	AF520172	
Tetrameles nudiflora R. Br.	Thailand: Central Botanical Garden, Saraburi, C. T. Philbrick & Wongpraseri 2272 (RSA)		U41502 [7]

1. Nickrent DL, Blarer A, Qiu YL, Vidal-Russell R, Anderson FE: **Phylogenetic inference in Rafflesiales: the influence of rate heterogeneity and horizontal gene transfer.** *BMC Evolutionary Biology* 2004*,* **4:** 40.
2. Thiele KR, Wylie SJ, Maccarone L, Hollick P, McComb JA: ***Pilostyles coccoidea*** **(Apodanthaceae), a new species from Western Australia described from morphological and molecular evidence.** *Nuytsia* 2008, **18:** 273–284.
3. Barkman TJ, McNeal JR, Lim S-H, Coat G, Croom HB, Young ND, dePamphilis CW. **Mitochondrial DNA suggests at least 11 origins of parasitism in angiosperms and reveals genomic chimerism in parasitic plants**. *BMC Evolutionary Biology* 2007, **7:**248.
4. Zhang L-B, Simmons MP, Kocyan A, Renner SS: **Phylogeny of the Cucurbitales based on DNA sequences of nine loci from three genomes: Implications for morphological and sexual system evolution.** *Molecular Phylogenetics and Evolution* 2006, **39:** 305-322.
5. Soltis DE, Soltis PS, Chase MW, Mort ME, Albach DC, Zanis M, Savolainen V, Hahn WH, Hoot SB, Fay MF, Axtell M, Swensen SM, Prince LM, Kress WJ, Nixon KC, Farris JS: **Angiosperm phylogeny inferred from 18S rDNA, rbcL, and atpB sequences.** *Biological Journal of Linnean Society* 2000, 133: 381-461.
6. Liston A, Rieseberg LH, Hanson MA : **Geographic partitioning of chloroplast DNA variation in the genus *Datisca* (*Datiscaseae*).** *Plant Systematics and Evolution* 1992, **181**: 121-132.
7. Rieseberg LH, Hanson MA, Philbrick CT: **Androdioecy is derived from dioecy in Datiscaceae: Evidence from restriction site mapping of PCR-amplified chloroplast DNA fragments.** *Systematic Botany* 1992, **17**:324-336.
8. Barkman TJ, Lim S-H, Salleh KM, Nais J: **Mitochondrial DNA sequences reveal the photosynthetic relatives of *Rafflesia*, the world's largest flower.** *Proceedings of the National Academy of Sciences of the United States of America* 2004*,* **101:**787-792.
